# Sex separation strategies: past experience and new approaches

**DOI:** 10.1186/1475-2875-8-S2-S5

**Published:** 2009-11-16

**Authors:** Philippos A Papathanos, Hervé C Bossin, Mark Q Benedict, Flaminia Catteruccia, Colin A Malcolm, Luke Alphey, Andrea Crisanti

**Affiliations:** 1 Imperial College London, Department of Biological Sciences, Imperial College Road, London SW7 2AZ, UK; 2 Medical Entomology Laboratory, Institut Louis Malardé, BP 30, 98713 Papeete, Tahiti - French Polynesia; 3 Entomology Unit, FAO/IAEA Agriculture and Biotechnology Laboratory, IAEA Laboratories, A-2444 Seibersdorf, Austria; 4 School of Biological Sciences, Queen Mary, University of London, Mile End Road, London, E1 4NS, UK; 5 Oxitec Ltd, Milton Park, Abingdon, Oxford OX14 4RX, UK; 6 Dept. of Zoology, University of Oxford, South Parks Road, Oxford OX1 2PS, UK

## Abstract

The success of the sterile insect technique (SIT) and other genetic strategies designed to eliminate large populations of insects relies on the efficient inundative releases of competitive, sterile males into the natural habitat of the target species. As released sterile females do not contribute to the sterility in the field population, systems for the efficient mass production and separation of males from females are needed. For vector species like mosquitoes, in which only females bite and transmit diseases, the thorough removal of females before release while leaving males competent to mate is a stringent prerequisite. Biological, genetic and transgenic approaches have been developed that permit efficient male-female separation for some species considered for SIT. However, most sex separation methods have drawbacks and many of these methods are not directly transferable to mosquitoes. Unlike genetic and transgenic systems, biological methods that rely on sexually dimorphic characters, such as size or development rate, are subject to natural variation, requiring regular adjustment and re-calibration of the sorting systems used. The yield can be improved with the optimization of rearing, but the scale of mass production places practical limits on what is achievable, resulting in a poor rearing to output ratio. High throughput separation is best achieved with scalable genetic or transgenic approaches.

## Background

Knipling first proposed releasing males to control populations of insects in sterile insect technique (SIT) programmes [1]. Due to the possibility of preferential mating between released sterile insects and the fact that released sterile females do not diminish populations, bisexual releases are far less effective and more expensive than male-only releases in introducing sterility into wild populations [2,3]. However, for the highly successful programme against the New World Screwworm *Cochliomyia hominivorax *both males and females had to be released [4], as there was - and still is - no sex separation system. For those agricultural pests in which females cause no damage, sex separation systems are not essential, but highly desirable in terms of increased efficiency.

For the early SIT programmes for mosquitoes, especially *Aedes aegypti*, sexes were separated using differences in pupal size, principally to increase efficiency. Since released sterile females may repeatedly feed on humans and thus contribute to disease transmission, tolerance for females in releases by programmes targeting vector species is likely to be much lower than for agricultural pests. For these vectors, SIT can, therefore, only be applied if some highly efficient way to exclude females is developed. It is surprising then, that in the earliest comprehensive description of SIT against public health vectors, developing sex separation methods is given almost no mention [5].

While the release of a small proportion of females may be acceptable for classical SIT, the effectiveness of a related technique, cytoplasmic incompatibility (CI), requires female-free releases in order to maintain its effect [6]. The CI effect depends on the reproductive incompatibility between released males and females of the wild target population. CI, manifested as male sexual sterility, is conferred to males by infections of maternally transmitted *Wolbachia*, a class of obligate bacterial endosymbionts that naturally induce CI or other reproductive anomalies, to increase the probability of their transmission in a given host population. Inadvertent escape of females infected with the same *Wolbachia *strain as the released males, could lead to chronic infections of the wild population, which would then no longer be controllable using the same male CI type. The risk associated with the release of contaminating CI females thus makes either female sterilization or the need for stringent, if not absolute, sex separation approaches even more critical.

There are methods under investigation that may require the release of females, and under certain conditions they may be deemed ethically acceptable. For example, when the disease in question occurs in relatively rare epidemics (e.g. dengue and yellow fever), transmission might not be significantly increased by intermittent release of small numbers of females, especially into a suppressed population. For population replacement strategies, which aim to spread a self-propagating heritable trait that diminishes vectorial capacity into the wild population, the release numbers required for successful delivery are typically estimated to be considerably lower than for SIT: this lower absolute number somewhat lessens the potential consequences of releasing females. Proof-of-principle has been demonstrated for one specific example in *Aedes aegypti*, based on the use of life-shortening *Wolbachia *[7]. Because of the strictly maternal inheritance of *Wolbachia*, use of this strategy would require the release of significant numbers of female mosquitoes. Therefore, each potential instance of release would need to be considered on a case-by-case basis.

The present article provides an overview of the various techniques used in the past, and being developed in the present, to separate sexes. It also describes and critically assesses the strength and limitations of current attempts to improve existing methods and to develop novel transgenic approaches for the large-scale production of male mosquitoes.

## Biological methods

Several inventive methods for sex separation of mosquitoes based solely on naturally occurring biological differences between males and females have been used. These have had varying degrees of success and were implemented at different scales.

### Mosquito pupal sexual dimorphism

In general, mosquito pupae are larger than larvae and female pupae are larger than male pupae. Size separation has therefore been a useful technique to separate pupae from larvae and males from females in the laboratory and in small factory settings. While pupae of most species can be separated efficiently from larvae using size or buoyancy differences [8], the stringency of sex separation of pupae is determined mostly by species-specific determinants and to a lesser extent by culture conditions. Both of these affect the degree of overlap between the sizes of males and females. In its simplest form, visual separation has been used to hand-select male *Culex quinquefasciatus *pupae [9], but this is of very limited use in the context of the large numbers of males needed for SIT. Major vector culicine male and female pupae differ in size more than anophelines so that the sexes of some culicine mosquitoes can be fairly efficiently separated by mechanical methods (discussed further below). Anopheline male and female pupae display much greater size overlap and size selection is not very discriminating.

Three types of mechanical devices to separate larvae from pupae, and sexes have been developed. Fay and Morlan [10] used adjustable glass plates to create a gap of uniformly diminishing width for larval removal and pupal size selection. Used very successfully with *Cx. pipiens*, the efficiency of the device was much lower for *Anopheles albimanus *[11]. While 170,000 males were mechanically separated using this device each day during the 1972 sterile male release study in El Salvador, the method only achieved an average of 86% male purity due to contamination by small female pupae. The original design was subsequently improved [12] and is still commercially available from the John Hock Company. A device using precisely positioned plates over a sluiceway has also been used for *Cx. pipiens *[13]. However, the size of the cephalothorax became greater on successive days of pupation and discrimination diminished. A similar system has been devised and is quite useful for culicines, but also has the same potential limitation of accommodating size change during the pupation period and according to culture conditions [14].

Sex separation in an ongoing SIT programme targeting *Aedes albopictus *in Italy is being performed at the pupal stage using standard metal sieves with a square-opening mesh through which males swim upward. With this system, small male pupae can be sorted to 97-99% purity, but recovery is inefficient with only 15-25% of total male pupae successfully separated and usable for irradiation and release [15]. While such low recovery is a handicap that can be compensated for under mass rearing conditions by the production of sufficient eggs, it translates into fixed programmatic costs (larger rearing facility, more staff, greater energy, food and water consumption, more waste to manage), which will diminish the economic viability of the control strategy. It is also possible that males selected by this process, being the smallest of the sample, may have reduced field performance, or that such selection may even lead to assortative mating in which wild females are selected to prefer the larger wild males. Mechanical separation at the pupal stage may also incur detrimental effects on the quality of the males produced, as was observed with mechanical sifting of Mediterranean fruit fly pupae. Damage to the indirect flight muscles significantly reduced the proportion of adults that could fly upon emergence (discussed in [16]). Although quite feasible, none of these methods have ever been fully mechanized (but see [8], Figure 1). It is probable that better size separation could be achieved if more consistent culture conditions existed. However, practical and biological limitations such as separation only at a late stage of development will limit the scope of their application.

### Exploiting behavioural differences

#### Blood-feeding

To improve sex separation, citrated bovine blood containing 0.05% malathion in condoms was fed to adults during the coastal El Salvador *An. albimanus *SIT programme [17]. The method removed 93% of females and required holding adults for three days before females would respond adequately to blood-feeding. Moreover, approximately 25% of the males were lost during treatment from contact with the contaminated blood or from other unknown factors [17]. The procedures used to package and transport males led to massive losses reducing the number of males available for release: 63% of sterile males typically died before reaching the release sites. While some improvements might be achieved with this method to improve female elimination and to reduce male insecticide exposure (e.g. attracting females to a surface coated with insecticide), the risks associated with insecticide handling in a mass rearing facility and the long holding time required before an effect is observed hardly make this approach the method of choice for large scale sex-separation.

#### Male swarming

One method based on adult behaviour has not been exploited: many mosquitoes mate in swarms consisting almost entirely of males. *Anopheles arabiensis *males display a strong swarming behaviour in prototypes of large cages currently tested at the International Atomic Energy Agency (IAEA) for factory scale production. This sex-specific behaviour could be exploited to suction large numbers of swarming males during their peak mating activity at dusk and dawn and may provide a rapid and fairly efficient sex separation strategy, but only in combination with other separation methods. It is possible that males with demonstrated swarming abilities in the factory are more likely to compete efficiently once released in the field. Like insecticide blood-feeding of females described above, this method would require at least 24 h before males began the necessary behaviour.

Sexing systems based solely on natural physical and behavioural differences between sexes are suitable for small-scale feasibility studies, but may represent a significant cost in a large-scale operational programme. Even then, to achieve the desired sexing stringency, more than one biologically-based sex separation method may need to be implemented sequentially at the pupal stage (e.g. sorting exploiting both the delayed female emergence and pupal size difference) and adult stage (e.g. sorting exploiting male/female differential behaviour such as blood-feeding).

While some of these methods have played useful roles, full scale area-wide integrated vector management (AW-IVM) programmes will require the development of more stringent and economically sound sex-separation techniques. Fortunately, several genetic and transgenic approaches have been devised to accomplish this. Some also provide greater economy because separation can take place early during development.

## Classical genetic methods

Conferring a selectable trait only to males as a means of *en masse *female elimination was proposed [18,19] and has been implemented by linking the selectable trait to a male-determining factor such as the Y chromosome of anophelines [20,21]. While constructing a candidate genetic sex separation strain (or "genetic sexing" strain, GSS) in the laboratory is sometimes simple, the development of a GSS able to meet the stringent requirements of stability, productivity and economy essential for successful long-term application in an SIT-based AW-IVM programme involves intensive research into the behaviour and genetic characteristics of the sexing strain and the species itself. Extensive knowledge of cytogenetics, mutation and strain analysis, markers and chromosome aberrations is available for many malaria mosquito vectors [22] and has been expanded greatly by more recent research (e.g. availability of whole genome sequences for *An. gambiae *and *Ae. aegypti*). However, this process remains time consuming since isolating a suitable selectable marker often requires extensive screening and good fortune. In mosquitoes, insecticide resistance is often detected and of relevance to control programmes, so resistant strains or alleles are often available for this purpose.

A classical *An. arabiensis *genetic sexing strain based on resistance to dieldrin was recently isolated at the IAEA and appears promising (unpublished). It was produced in a manner that was previously successful for *An. gambiae *[20], *An. arabiensis *[23] and *An. albimanus *[24]. It has achieved high discrimination (> 99.5% males), long-term stability and reliability required for safe and nearly female-free releases. However the mating competitiveness of the strain is still unknown.

To date, GSS have been developed for 20 insect species [25] but for only two of these, *An. albimanus *and the Mediterranean fruit fly *Ceratitis capitata*, have the strains been developed to the point where they could be mass-reared at levels required for AW-IPM programmes integrating the SIT [26]. Only in the Mediterranean fruit fly was the separation system improved sufficiently for truly large-scale application over extended periods of time. In this strain, sex separation is based on a mutation causing temperature sensitivity of female embryos. Use of this strain has contributed to releases being on a far greater scale than for any other programme and highlights the value of robust sex-separation methods.

Secondary visible markers that can be used in conjunction with the selectable marker and which are also linked to the male determining chromosome are also useful for stock maintenance and detecting the presence of recombinant individuals: The *white pupae *(*wp*) mutant in the Mediterranean fruit fly is linked to the *temperature sensitive lethal *(*tsl*) mutation and male determining chromosome and thus provides a visual indicator for the efficiency and stability of the GSS, as male pupae are brown and female pupae are white [26]. This tight association between the *tsl *and *wp*, together with the prolonged developmental time of *tsl *mutant homozygotes compared to wild types or heterozygotes, allows for a highly efficient removal of individuals arising from destabilising recombination events. Since large-scale mass-rearing conditions introduce innate difficulties in maintaining GSS stability, when visible mutations such as *wp *are available, a Filter Rearing System (FRS) can be used. Surplus insects from a small "mother" colony are fed into the unidirectional high-density population destined for release. The "mother" colony is maintained at low-density conditions, primarily to reduce selection pressures, allowing for small-scale screening and thus highly-efficient elimination of recombinants [27]. This simple characteristic also provides an obvious indicator of operational sex-separation failures for production personnel. Compared to mechanical approaches, GSS strains display multiple advantages: 1) female elimination is more thorough and predictable. *Anopheles albimanus *pupae produced during the five-week period of the SIT programme conducted on the Pacific coast in El Salvador were 99.9% males with an average adult emergence of 90% and for the Mediterranean fruit fly over 99% males are being produced at production levels of over two billion males per week. 2) When females can be eliminated by exposing eggs to insecticide or high temperature, significant savings can be made in production and release costs, and it provides 3) greater flexibility of release methods - e.g. release of pupae rather than adults.

All of the above approaches require either a physical characteristic that is intrinsic to a species or the *ad hoc *generation of novel genetic tools for each species independently. Unfortunately, the classical approach to developing a GSS can be elusive or serendipitous, is usually a long process, and its success cannot be guaranteed.

## Transgenic methods of sex separation

Similarly to classical systems, transgenic sexing systems encompass numerous technological approaches which, whilst often diverging in method, have been proposed or are being developed for the goal of efficient sex separation. Compared to biological and genetic methods for sex separation, transgenic methods offer the key advantage that the available sexing systems are not entirely based on strain-specific naturally occurring or *ad hoc *developed biological or genetic variations between the sexes. In almost all cases there is no *a priori *reason to expect that sexing technologies developed for one species may not be transferable to a number of important pest or vector species with little modification of the of the transgenic constructs. In some instances, modifications to individual components such as endogenous species-specific promoters, or sex-specific splicing cassettes and lethal effectors may increase the efficiency of the sexing construct, whilst simultaneously avoiding the potential detrimental effects of accidental species transfer.

### Fluorescent sorting

Sex-specific expression of transgenes has been achieved for the Mediterranean fruit fly with the generation of transgenic strains harbouring selectable markers on the Y chromosome [28]. The selectable marker used in this GSS was a fluorescent protein, and sex separation was achieved by scoring newly-hatched larvae for the fluorescence trait. In two transgenic strains, Y chromosome-linked conventional transposon constructs harbouring the fluorescent marker were useful in producing small scale male-only transgenic populations. Expression of the fluorescent marker was considerably weaker than that typically observed for equivalent insertions on autosomes, presumably due to the effect of the surrounding heterochromatic environment, however sex-specific expression was sufficiently robust to permit sexing in several independent Y-linked insertions.

Rather than using sex-linked expression, alternative selection systems based on the use of sex-limited expression of transgenes have been produced. The first example of such a strategy was reported for *An. stephensi*, where a sperm-specific promoter from the *An. gambiae β2-tubulin *gene was used to drive expression of a fluorescent protein [29]. Sex separation was accomplished using the mechanico-optical COPAS sorting system [30]. Using conservative instrument settings to maximize transgenic sorting accuracy, female-free sex separation was achieved by this method, however the percent male recovery was not reported. This remains the only transgenic sexing system so far published for mosquitoes. One drawback is that the male-specific promoter used is expressed late during spermatogenesis in the testes and is therefore only visible in the latter larval stages. The construct expressing the transgenic sexing trait system has since been successfully transferred to other relevant species including *Ae. aegypti *[31], *An. gambiae *[32], *An. arabiensis *(J. Thailayil, pers. comm.) and *C. capitata *[33], although the accuracy of sexing using fluorescence-based sorting has not been reported for these species. In general, all fluorescence-based sorting systems suffer the disadvantage that each larva needs to be individually examined and sorted. Instruments for automatic fluorescence-based sorting exist, but to achieve sex separation on the large scale required for AW-IVM programmes, these instruments will certainly require additional development in order to reduce cost, and to increase throughput and discrimination.

### Female elimination

Sex separation using sex-limited transgenic constructs could be more efficiently achieved by combining positive selection, for example insecticide resistance, with male specific or male biased expression. A testis-specific promoter such as *β2-tubulin *however, is unlikely to be useful to express a chemical selection system such as insecticide resistance as expression in the testes only will not likely confer resistance to the whole mosquito. Male-specific promoters that are expressed ubiquitously have yet to be isolated in any current or candidate SIT-species. Alternatively, sex separation could be achieved by negative selection against females using synthetic conditional female-specific lethality traits, whereby females can be selectively eliminated by changing the rearing conditions of insects that will be released. These strategies have the advantage of population treatment, so that sex-separation can be performed on large numbers of larvae simultaneously. Negative selection systems have been built in *Drosophila melanogaster *using female-specific promoters [34,35] or using sex-specific alternative splicing in *D. melanogaster *and the Mediterranean fruit fly [36]. In each case these systems have been constructed using the "tet-off" conditional gene expression system [37,38], so females survive in the presence of low concentrations of tetracycline, or a suitable chemical analogue thereof, but die in the absence of tetracycline. Such systems also provide a degree of biocontainment, as such females cannot breed in the wild, and it has also been proposed that such systems can be used for 'genetic sterilization' in place of radiation-sterilization [35,39-41].

### Manipulation of sex determination and sex ratio

Another interesting method for eliminating females from the population prior to release is the conditional manipulation of insect sex determination or sex ratio. In vector species considered for SIT, sex is initially determined by genetic constitution, and more specifically by the inheritance of one of the two paternal sex chromosomes. As a general rule (although this is not always the case), inheritance of a paternal X chromosome leads to the initiation of a sex determination cascade that produces females, whilst inheritance of the male-determining chromosome (the Y chromosome in *Anopheles*) will result in the initiation of male determination cascade [42]. Natural sex-ratio distortion systems occur in some species (e.g. *Ae. aegypti *[43]), though the molecular basis of these is not well understood. Progress towards the development of a synthetic sex distorter has been reported for a transgenic *An. gambiae *strain [44]. Expression of an endonuclease that targets sequences located exclusively on the X chromosome during spermatogenesis led to significantly increased Y chromosome transmission to progeny of transgenic males. However, all embryos fertilized by sperm from these males were inviable; the authors attributed this to the endonuclease targeting the maternal X chromosome after fertilization.

For a number of important pest and vector insects, several genes that may function in the sex determination pathway have been identified, mostly based on orthology relationships with *Drosophila*, whose sex determination cascades are understood in great detail and used as a model. Often, due to the intrinsic high rate of evolution of these pathways, genes found by this method are not functionally related to their *Drosophila *counterparts, such as *Sxl *[45] and the X:A ratio versus the male-determining Y-linked primary signal [42]. However, for some of these genes, where sequence and functional relatedness have overlapped, sex separation strategies that manipulate their function are being developed that when perfected will represent the ultimate in "sex separation" efficiency. In the Mediterranean fruit fly conversion of genetic XX females into males by transient interference of the *transformer *gene using injected dsRNA caused complete sexual conversion of both germline and somatic tissues in adult flies. The resulting XX male individuals could mate, transfer sperm and remained fertile [46]. A transgenic line harbouring an inverted repeat driven by a heat-shock promoter corresponding to the *Cctra *produced male only progeny (95% males and 5%intersexes) following heat pulses during development [47].

A limitation of transgenic strategies manipulating insect sex for the purpose of male only releases is the potential specificity to the species that they are initially developed for, in which case transfer to other important species may be complicated to achieve. For example, the ability to directly transfer the medfly female-to-male converter technology to African mosquitoes will likely be limited by a failure to identify the species counterpart for the ambiguous *transformer *gene, although the central technology for transient RNA interference may be widely used. Inversely, given that the ribosomal RNA genes targeted by the aforementioned *An. gambiae *synthetic sex ratio distorter system are only specific to the X-chromosome in a few *Anopheles *species, this strategy as well may be limited to candidate SIT species which share this genetic arrangement with *An. gambiae*.

## Conclusion

Before transgenic technologies can be brought to the field, suitable modifications may need to be introduced into these strains to ensure performance under highly demanding mass rearing conditions and to address the perceived risks related to their use in open environments. As much as possible, female elimination technologies should be developed in generic ways that do not restrict their use to a particular species, location, or application. These technologies would be most widely applied if they were developed without a particular species in mind and satisfied the needs of projects that are already funded or which would anticipate clear future needs if they were successful. Individually or together with other components of SIT, sex separation processes should be useful for fertile transgenic release, classical SIT or cytoplasmic incompatibility population suppression.

In summary, several methods exist or are in development for sex-separation of mosquitoes. The choices that are available are much less limited if the location in which the technology will be applied is one in which the release of transgenic insects is acceptable. In this case, several flexible methods are becoming available. Even in their absence, physical and classical genetic methods are available that - while they are often less effective and more difficult to create - do not present an insurmountable hurdle to implementation of area-wide control using SIT. While the initial effort to create them may require several labour years, this is a small investment relative to the cost of establishing production and conducting mass-rearing and release. Priority should also be given to developing measures of male competitiveness and determining the genetic, behavioural, and physiological components that control it. In the context of mosquito vectors, the creation of sex-separation strains by any means is essential.

## Outcomes for sex-separation technology - 7S

Seven key outcomes (the "7 Ses") were extracted from the various sex separation technologies presented in this article.

### Small

Early removal of females during development frees resources for production of more males and reduces the cost of males released.

### Simple

Factory production methods are most robust when simple processes are applied. An *en masse *selection through e.g. heat and chemical exposures will likely be more consistently applied and adopted over methods involving individual mechanical sorting, inter-strain crosses, or the prolonged use of labile chemical treatments.

### Switchable

Because a broodstock colony must be maintained for production of males, the mechanism that eliminates females must be conditional.

### Stable

Unstable sex separation methods require frequent stock re-purification and diligent monitoring, which in the long run, are not acceptable for a mass rearing operation. Any system will need to be monitored, but a low frequency and accumulation of undesirable exceptional individuals reduces the reliability and predictability of mass rearing and sexing, adds cost to production and potentially hazards upon release. Efficient mass rearing will likely required the use of stabilizing strategies, such as the FRS.

### Stringent

How much female contamination is acceptable? Nuisance biting and potential disease transmission by released females must be considered. The *An. arabiensis *GSS based on resistance to dieldrin achieved at best 99.5% female elimination (unpublished), whilst *tsl/wp Ceratitis *GSS releases achieve 99.75% [26]. Assuming an over-flooding ratio of 100, the resulting female population would increase during the release generation by 100% and 50% respectively. Would such increases in the female population be acceptable in classical SIT programmes targeting mosquito vectors?

### Sexy

Sterile males must arrive in the field able to compete for wild females. This means that the selection method must not create inordinate reductions in male competitiveness. If introgression of genetic material into the sexing strain is necessary to increase competitiveness, vital sexing components within a large chromosome tract that suppresses recombination will be less amenable to such an effort than a single locus containing a transgene.

### Sellable

Will the technology be acceptable in the target release area? Can one obtain political support for implementation of the specific technology? While it is too early to be dogmatic, transgenic systems may be more readily accepted in disease-endemic areas as speculative risk scenarios are less weighty when the real prospect of continuous disease transmission is considered in comparison to areas where the vector is merely a pest. In 'pest-only' settings, the prospect of transgenic insect releases might reduce or eliminate national and local support. Applications of some genetically modified insects will be facilitated by the fact that under the North American Plant Protection Organization standards [48], sexual sterility is considered a means to accomplish confined release. If similar standards are applied to mosquitoes, the number of areas in which releases are permissible may increase.

## Competing interests

LA is an employee and shareholder of Oxitec Ltd., which is developing technologies related to the subject of this article. The other authors declare no competing interests.

## Authors' contributions

PAP, HCB MQB coordinated the writing and wrote much of the discussion on sex separation outcomes. AC, FC, LA and PAP contributed the molecular sex separation discussion. CAM contributed the section on classical sex separation. All authors read and approved the final manuscript.
